# What makes a good clinical student and teacher? An exploratory study

**DOI:** 10.1186/s12909-015-0314-5

**Published:** 2015-03-10

**Authors:** John Goldie, Al Dowie, Anne Goldie, Phil Cotton, Jill Morrison

**Affiliations:** 1 Glasgow University Medical School, Glasgow, Scotland UK; 2 Tom Allan Centre, Glasgow, Scotland UK; 3 College of Medicine and Health Sciences, University of Rwanda, Kigali, Rwanda; 4 College of Medical, Veterinary and Life Sciences, University of Glasgow, Glasgow, Scotland UK; 5 Academic Unit of General Practice and Primary Care, University of Glasgow, 1 Horselethill Road, Glasgow, G12 9LX Scotland UK

**Keywords:** Good clinical student, Good clinical teacher, Undergraduate medical education

## Abstract

**Background:**

What makes a good clinical student is an area that has received little coverage in the literature and much of the available literature is based on essays and surveys. It is particularly relevant as recent curricular innovations have resulted in greater student autonomy. We also wished to look in depth at what makes a good clinical teacher.

**Methods:**

A qualitative approach using individual interviews with educational supervisors and focus groups with senior clinical students was used. Data was analysed using a “framework” technique.

**Results:**

Good clinical students were viewed as enthusiastic and motivated. They were considered to be proactive and were noted to be visible in the wards. They are confident, knowledgeable, able to prioritise information, flexible and competent in basic clinical skills by the time of graduation. They are fluent in medical terminology while retaining the ability to communicate effectively and are genuine when interacting with patients. They do not let exam pressure interfere with their performance during their attachments.

Good clinical teachers are effective role models. The importance of teachers’ non-cognitive characteristics such as inter-personal skills and relationship building was particularly emphasised. To be effective, teachers need to take into account individual differences among students, and the communicative nature of the learning process through which students learn and develop. Good teachers were noted to promote student participation in ward communities of practice. Other members of clinical communities of practice can be effective teachers, mentors and role models.

**Conclusions:**

Good clinical students are proactive in their learning; an important quality where students are expected to be active in managing their own learning. Good clinical students share similar characteristics with good clinical teachers. A teacher’s enthusiasm and non-cognitive abilities are as important as their cognitive abilities. Student learning in clinical settings is a collective responsibility. Our findings could be used in tutor training and for formative assessment of both clinical students and teachers. This may promote early recognition and intervention when problems arise.

## Background

What makes a good clinical student is an area that has received little coverage in the literature [1]. It is particularly relevant as recent curricular innovations have resulted in greater student autonomy in managing their own learning [2]. Glasgow University’s medical curriculum has undergone such curricular change with the introduction of a learner-centred, problem-based curriculum and the embracing of an outcome-based approach to learning [3]. As part of a larger research study, looking at how students form their professional identities, we were interested in our students’ and tutors’ views on what makes a good clinical student.

Teaching and mentoring medical students and junior doctors is considered “Good Medical Practice” [4]. Doctors who undertake teaching and supervision should also develop the skills, attitudes and practices of a competent teacher [4]. Recent literature reviews [5-7] have identified a degree of consensus on what characterises a good medical teacher:

Clinical knowledge, clinical and technical competence, positive relationships with students, effective communication skills, enthusiasm.

Many of the papers identified, however, were essays or surveys of students, residents or colleagues [5]. We also wished to explore in more depth students’ and tutors’ perspective on what makes a good clinical teacher.

We hoped the findings would inform and promote reflection by Medical School staff, management and students, and contribute to the wider literature.

## Methods

We decided to use individual interviews with educational supervisors, clinical teachers responsible for mentoring students and overseeing their clinical education, and focus groups with senior students from years 4/5 (a two-year clinical rotation). It was felt that the use of focus groups would promote a feeling of safety, encourage participation and help generate more realistic accounts of what students’ think [8]. This had to be balanced against the likelihood of eliciting conventional or conforming group-think, which was felt less likely as the group was homogenous with no obvious power differentials among members.

### Ethical approval

Ethical consent for the study was obtained from the College of Medical, Veterinary and Life Sciences Ethics committee and the Greater Glasgow and Clyde National Health Service Local Research Ethics Committee.

### Participants

A purposive sample of ten educational supervisors, stratified in terms of the range of specialties involved in teaching and whether the supervisor worked in a Glasgow teaching hospital or in the 19 West of Scotland hospitals that deliver 30% of clinical teaching at Glasgow University, was proposed. Hospital sub-deans were approached to help identify suitable participants. Unfortunately due to a poor initial response the researchers also made individual contact with potential participants. Our participant group worked in a range of specialties and settings (Table 1). Informed consent was obtained from each supervisor.

**Table 1:** **Characteristics of the Educational Supervisors interviewed**

Gender	8 Male
	2 Female
Hospital	6 Glasgow Teaching Hospitals
Base	3 District General Hospitals
	1 Psychiatric Hospital
Specialty	7 Medical specialties
	2 Surgical
	1 Psychiatry

Three focus groups were to be run with purposive samples of year 4/5 students. Due to poor student response a convenience sample of three groups of 5 to 6 students participated. They were representative of year 4/5 students in Glasgow in terms of age, gender and holding previous and intercalated degrees. Informed consent was obtained prior to participation.

The interviews were conducted by AG a researcher external to the Medical School whose background is in nursing and counselling research. The interviews were semi-structured. AG and AD, our qualitative researcher, met on a number of occasions prior to the interviews to produce a broad topic guide of the issues to be covered based on the aims of the study. A conversational style was adopted with participants encouraged to respond freely. The topic guide was refined following reflection on the initial interviews.

The first and third focus groups were conducted by JG a researcher in the School of Medicine who had no previous contact with any of the participants. The second was conducted by PC, who was Associate Dean for Student Welfare at the time. The broad topic guide was again used. Prior to the groups taking place, the moderators met with AD and AG to help maximise uniformity of the moderator approach, which was to keep intervention to a minimum. However, the moderators were encouraged to play devil’s advocate where necessary if there was a perceived threat of group think.

### Analysis

The interviews and focus groups were audio-taped and transcribed. Although review of the transcripts and discussion of themes was ongoing, the main process of analysis was carried out when data collection was completed. A “framework” technique developed by the National Centre for Social Research was used [9]. This was considered an appropriate approach in a study where some of the research questions were predetermined and which was geared towards generating practice-orientated findings. Analysis was performed by JG. The first step involved familiarisation with the data. The next steps involved thematic analysis to develop a coding scheme. The data was then indexed and charted with comparison both within and across the interviews. Discomforting data was examined, for example one supervisor had not witnessed much interaction between students and health care professionals. Given the position of the moderator, PC, the dynamic of his focus group was examined to determine if it had been adversely affected. No discernible difference was found in the content generated in this focus group compared with the other two. There were only a few occasions where the moderators played devil’s advocate. In mapping and interpreting the data, locating the empirical findings from within the wider literature on professionalism, social learning and identity, helped make sense of the data.

The last two interviews and the third focus group generated no new data. Data saturation was considered to have been reached.

The analytical process and findings were reviewed throughout by AD, AG and PC.

## Results

The themes identified by the interviews and focus groups are shown in Table 2. The numerical counts are intended to convey a sense of the distribution of themes across interviews and focus groups.

**Table 2:** **Themes identified by interviews and focus groups**

Characteristics of a good clinical student	Characteristics of a good clinical teacher
*Teachers*	*Teachers*
Participates in learning/good attendance – 10	Act as positive role models - 10
Proactive – 5	Develops relationship with students - 6
Enthusiastic – 8	Respects students - 5
Mature/confident/not afraid to ask for help – 8	Cares about students - 4
Knowledgeable/skills competence – 7	Promotes students’ participation in communities of practice - 6
Communication skills – 6	Makes time to teach -5
Takes responsibility for learning/independent – 5	Provides one-to-one teaching - 4
Does not let exam pressures interfere with performance on attachment −5	Interested and committed to teaching - 5
Caring of patients/genuine – 4	Provides feedback - 5
Sense of vocation – 3	Relevant to curricular stage - 2
Team player – 3	
Able to recognize position in hierarchy - 1	
Should conform to norms of dress – 7	
Avoid jeans, earings/piercings, bare midrifts/sexualized appearance	
Should become fluent in language of medicine/bilingual in being able to adjust language when talking to patients – 7	
***Students***	***Students***
Participates in learning/good attendance – 3	Act as positive role models – all 3 groups
Proactive – 3	Develops relationships with students - 3
Enthusiastic – 3	Enthusiastic/appears to enjoy teaching/confident - 3
Knowledgeable – 3	Knowledgeable/technically competent - 3
Able to take risks in a safe environment – 3	Makes time to teach - 3
Offer peer support – 3	Provides relevant learning experiences - 2
is promoted by shared experience and seen as an investment in the future	Promotes students’ abilities to take risks in safe environments - 2
Confident – 2	Supportive - 3
Aware of limitations – 2	Encouraging- 3
Team player – 2	Treat students with respect - 3
Able to take criticism – 2	Provide honest constructive feedback - 3
Should conform to norms of dress – 3	Provides feedback consistent with curricular stage - 2
Students aware of overt pressure to conform to norms of dress – 3	Genuine with patients, students and colleagues - 3
Avoid casual dress, piercings, sexualized appearance – 3	Good interpersonal skills - 2
Promotes identification as medical student – 2	Team player - 3
Taken more seriously by ward staff −2	Avoids humiliating students - 3
Can hide behind clothes/make oneself look older – 2	Has developed teaching skills - 2
Can become less conformative as move up hierarchy – 2	Appears to have a true vocation - 2
Changing clothes is a method of switching off – 2	Ability to listen - 2
Should become fluent in language of medicine/bilingual in being able to adjust language when talking to patients – 3	Sense of humour - 1
**Junior doctors and other Health Care Professionals as role models and mentors**	
***Teachers***	***Students***
It is important for students to develop relationships with junior doctors who can act as role models and mentors – 5	Junior doctors are able to empathise more with students - 3
Students identify with junior doctors more easily – 4	Can be more welcoming
It is important for students to develop relationships with other health care professionals – 5	More approachable
Helps students learn about teamwork	More able to form relationships with students
	Other Health Care professionals can be effective teachers and mentors - 2
	Nurses provide mentoring for developing practical skills
	Useful source of practical knowledge
	Important to develop relationships with HCPs to facilitate teaching
	Recognition of importance of establishing relationships to facilitate learning
	HCP, especially nurse teaching helps boost confidence and makes student feel part of team

### What makes a good clinical student

Students considered to be good, proactively participate taking every opportunity to learn. They are considered knowledgeable, mature, confident, team players who are able to take to criticism. Poor students, in contrast, attend less frequently and when they do, tend not to participate to the same extent. They appear to supervisors as anxious and scared of patients’ illnesses and seem afraid of taking responsibility.

“(Good students) they will come and ask for computer passwords to get access to results to help on the ward round tomorrow. That would give me a signal that they’re keen to learn and get experience of what real life work is. They are trying to be helpful and (contribute) to the team…they stay behind at night and give the resident a hand…their case histories (are more insightful)”.

(Supervisor interview A)

“(A good student) is intelligent, but it is on display, they’re interested, ask questions, they want to know things…that stimulates me (in turn) and I get right into it…A bad student is the one who turns up late, or not at all, they give the impression they’re only there to tick the box. I mean the good student engages with you (has good eye contact) verses the (bad student) who stares at the ceiling or looks at the floor and you’re lucky if you get three syllables out of them”.

(Supervisor interview J)

“The best ones have good background knowledge and are able to prioritise it and apply it quickly…they grasp the important things quickly…(Poor students) haven’t put the time in to (acquire) the same knowledge…they can’t prioritise the important bits of information from the less important, for example picking out the relevant bits from the history and bringing it together in a diagnostic solution….(The best students) also approach patients like fellow human beings…they can relate to patients and laugh with them”.

(Supervisor interview I)

“(Poor students) turn up late and clock off early. If they can’t manage (for example) to site a venflon and its getting near when they finish they don’t take responsibility and get someone else to do it, they just leave. They have no sense of vocation”.

(Supervisor interview B)

“(Poor students) when asked (during ward teaching) to take a history and examine a patient get very anxious about it. Their a bit scared of them I think because (the patients) are ill and I suppose there’s a huge anxiety of thinking in a few years time that (they are) going to (be responsible)”.

(Supervisor interview C)

“(Bad student)…unprepared, lazy, doesn’t take the initiative…(Good students) take the initiative, if you do you can have endless learning opportunities…just present yourself with your badge and you can, for example, do pathology for a day…even better if a consultant takes you under their wing. I got one who let me scrub in and I got to assist at lots of operations”.

(Student focus group 1)

“(Good student) needs to be intelligent, dedicated, but at the same time you need to have a perspective on what your learning…self awareness of your competency…Also it’s your responsibility (to develop knowledge and skills), you have to put yourself out there, for example find a doctor to show you how to take blood”.

(Student focus group 3)

They are expected, by both their teachers and their peers, to dress conservatively and become fluent in medical terminology while retaining the ability to communicate effectively and be genuine when dealing with patients. They do not let exam pressure interfere with their performance during their attachments.

“Some of them don’t know what a hemicolectomy means…they use terms like wound in the tummy…while its important to be (able to communicate effectively with patients)…when students use professional language it must be correct…It allows everyone (in the profession), from Land’s end to John O’Groats, from Florida to Alaska, to understand (what you’re talking about)”.

(Supervisor interview B)

### What makes a good clinical teacher

The importance of the clinical teacher as a positive role model and mentor was emphasised by both students and supervisors:

“He was just himself with (his) patients… he was sitting forward, nodding, doing everything it says in the book, but really empathic. I think when you can do that and your patients like you and respect you then I think you are a doctor rather than someone trying to be a doctor”.

(Student focus group 1)

“I had a (teacher) who took me through a chest X-Ray and I don’t think I’ll ever forget it …he compared it to looking at a tree…you need to look at all the bits you might forget first like the diaphragm, that’s the roots…then the vertebrae, that’s the trunk, and then the main branches, that’s the ribs…analogies like that, do help”.

(Student focus group 3)

“When (students are) shadowing me (it is possible) to model a sense of vocation…if my list has finished early and a colleague’s is running late (as an example of teamwork) I’ll take cases from him and do them for him so that (the cases) are managed appropriately and (promptly)”.

(Supervisor interview A)

“I had this student who performed badly in her clinical exam …I took her aside and said ‘let’s just think about what happened’…inside five minutes she was in tears and we worked through to the fact she had major exam nerves …and I managed to point her in the direction of psychological help. Later I bumped into her after her (finals) OSCE… she was smiling and did fine”.

(Supervisor interview J)

Students could also learn from negative role models

“I think respect‘s a big thing. I’ve been on wards where the doctor might be skilled (technically) but doesn’t have a good manner with (patients and staff)…You can tell the nurses don’t like them and make jokes about them behind their backs…I want to have the respect of my colleagues as well as be capable of (doing) my job well”.

(Student focus group 3)

Good clinical teachers are considered to be skilled communicators who are knowledgeable, committed, enthusiastic and genuine. They should have good teaching skills. They take time to develop relationships with students and encourage students’ participation. They provide honest, constructive feedback relevant to students’ curricular stage. They are observed to exhibit the same characteristics during their clinical work:

“I think it’s (teachers) who take time with you and treat you as an equal…people with a superior level of knowledge that can impart that to you well…people that seem to relate to patients and other team members very well. Someone you feel you have confidence in and would like them to be your doctor”.

(Student focus group 3)

“A bad teacher …harasses and berates you in front of other members of staff…doesn’t give you constructive criticism…in order to learn you have to take risks, to ask questions and to question others and it makes you not to want to…You have to instil (students with) confidence, you have to make them feel they’re actually able to do it, emulate what’s in front of them. When you humiliate students…it’s counterproductive they just regress (your) not inspired to go away and learn (from your mistakes). A good teacher…they’ve been training doctors for ages and pick up my strengths and weaknesses without me saying it and they say ‘ok you’re quite good at this but let’s work on that’”.

(Student focus group 1)

Junior doctors can be effective teachers and mentors. They are perceived by students as often being more approachable and empathic. Other health care professionals, particularly nursing staff, could also be effective teachers and mentors. These relationships are promoted by students’ proactive participation and the development of interpersonal relationships.

“There was this junior doctor who was really efficient and on top of things (and helped us when asked). What was (just as) important was she always had a big smile for everybody, even when things were getting stressful she kept it together”.

(Student focus group 3)

“Nurses are really good sources of information. They know the routines, medications and normal practices far better than we do (or even) the junior doctors”.

(Student focus group 3)

“Sometimes it’s the other health care professionals that are more receptive to students. I (went) to some physio sessions and that’s where I learned most of my orthopaedic examinations…you find a person who takes you under their wing…boosts your confidence as well as pushing you to learn…it all comes back to that whole communication thing and forming relationships”.

(Student focus group 1)

## Discussion

The study was limited by resource and the inaccessibility of students and supervisors due to the wide geographical range of teaching units used for clinical teaching at the University of Glasgow. The purposive sample of supervisors had to be augmented by approaching individual tutors and a convenience sample of students had to be used. Despite the recruitment problems, and not using the same moderator for all three focus groups, meaningful data were considered to have been produced. These data can be used to inform an observational study of clinical settings, which would strengthen the validity of the findings.

Good students were viewed, by both supervisors and students, as being enthusiastic and motivated. They were considered to be proactive in seeking out relevant learning experiences and were observed by supervisors as being visible in the wards. This is an important quality where students are expected to be active in managing their own learning. They appear confident, knowledgeable, able to prioritise information, flexible and competent in basic clinical skills by the time of graduation. The need to be proactive, while being adaptive, however is another example of medical education’s blending of incompatible norms [10,11]. Good students are considered by supervisors and students to be compassionate, able to communicate with, and be respectful of, patients and colleagues. They were also observed to be genuine when interacting with patients and colleagues. They are not afraid to seek help when necessary and are aware learning is a lifelong commitment. They are felt to have a sense of vocation. Poor students on the other hand were noted, by both supervisors and students, to attend less frequently and when they do, tend not to participate to the same extent. They appear anxious and scared of patients’ illnesses and seem afraid of taking responsibility. They appear not to be as interested, although some supervisors reflected this was not the case at their medical school selection interviews. They were viewed by supervisors as making less effort, being less knowledgeable, less able to prioritise information and having less sense of their future roles. Supervisors felt they also interacted less well with ward team members. Validation of identity by patients and members of communities of practice was found to promote professional inclusivity [12]. However, students who show unwillingness to participate, or are on the margins may have their identities’ disconfirmed and may be deprived of learning opportunities [13]. Non-participation among students may, however, be a way of resisting the “normalizing technologies” of professional socialization [14]. This should be considered and explored by teachers.

Our findings could be used for clinical students’ formative assessments and help evaluate their effectiveness as self-directed learners in clinical settings. They could help flag up concerning behaviours at an early stage enabling appropriate intervention. They could also be useful for tutor training.

The findings on what characterises a good teacher are consistent with other studies and reviews [5-7,15,16]. They exhibited the characteristics highlighted in the literature on effective role models; high standards of clinical competence, excellence in clinical teaching skills and humanistic personal qualities [17]. The importance of non-cognitive characteristics such as inter-personal skills and relationship building was particularly emphasised by both students and supervisors. Our findings are also consistent with Dornan et al’s study [18] that effective clinical teachers simultaneously support and challenge students in ways that builds competence and confidence. Effective teachers are those who stretch students, using a repertoire of teaching and learning approaches, but who do not take students too far outside their Vygotskyian Zones of Proximal Development (ZPD). To be effective, teachers need to take into account individual differences among students, and the communicative nature of the learning process through which students learn and develop [19,20]. Good teachers were noted to promote student participation in ward communities of practice. The best teachers were considered to exhibit similar characteristics in their clinical work and were considered genuine when interacting with patients and colleagues. This reflects Goffman’s [21] distinction between convincing and unconvincing performances.

Teachers who were felt to be poor were found to push students beyond their ZPDs, having little awareness of students’ learning needs and individual characteristics, with a tendency to humiliate students when they exhibited lack of knowledge or skills. This has the effect of marginalising and de-motivating students. It reflects a disconnection between what students were taught in the classroom about professionalism and what they experience in the ward setting [21]. Teachers exhibiting these forms of behaviour are considered by students to be unprofessional and are recognised as negative role models. A particular characteristic of these teachers is a perceived lack of interpersonal skills resulting in poor relationships with students, patients and colleagues. The abuse of power exercised by such teachers can have negative consequences for student learning.

Our findings could again be used in tutor training and as a basis for formative assessment of tutors by both colleagues and students.

Other members of the clinical communities of practice can be effective teachers, mentors and role models. Students’ participation in the communities of practice is pivotal. Students are aware of the need to foster relationships with their teachers, other doctors and members of the ward team, which is recognised as an important investment in their learning. Student learning in clinical settings should be seen as a collective responsibility.

## Conclusions

- Supervisors and students see good clinical students as being proactive in their learning; an important quality where students are expected to be active in managing their own learning.
- Good clinical students seem to share similar characteristics with good clinical teachers
- Student learning in clinical settings is a collective responsibility.
- A teacher’s enthusiasm and non-cognitive abilities are as important as their cognitive abilities.
- Our findings could be used in tutor training and for formative assessment of both clinical students and teachers. This may promote early recognition and intervention when problems arise.
